# Supplementary material for: Multi-scale integration of human brain vascular and CSF proteomes reveals biomarkers of cerebral amyloid angiopathy linked to Alzheimer’s disease risk
Source: medRxiv. 2025 Oct 16:2025.10.08.25337413. Preprint. [Version 2] doi: 10.1101/2025.10.08.25337413 (PMC12633097; doi:10.1101/2025.10.08.25337413)
Supplement: 1 [file NIHPP2025.10.08.25337413V2-supplement-1.pdf]

medRxiv preprint doi: https://doi.org/10.1101/2025.10.08.25337413; this version posted October 16, 2025. The copyright holder for this preprint (which was not certified by peer review) is the author/funder, who has granted medRxiv a license to display the preprint in perpetuity.
It is made available under a CC-BY 4.0 International license .

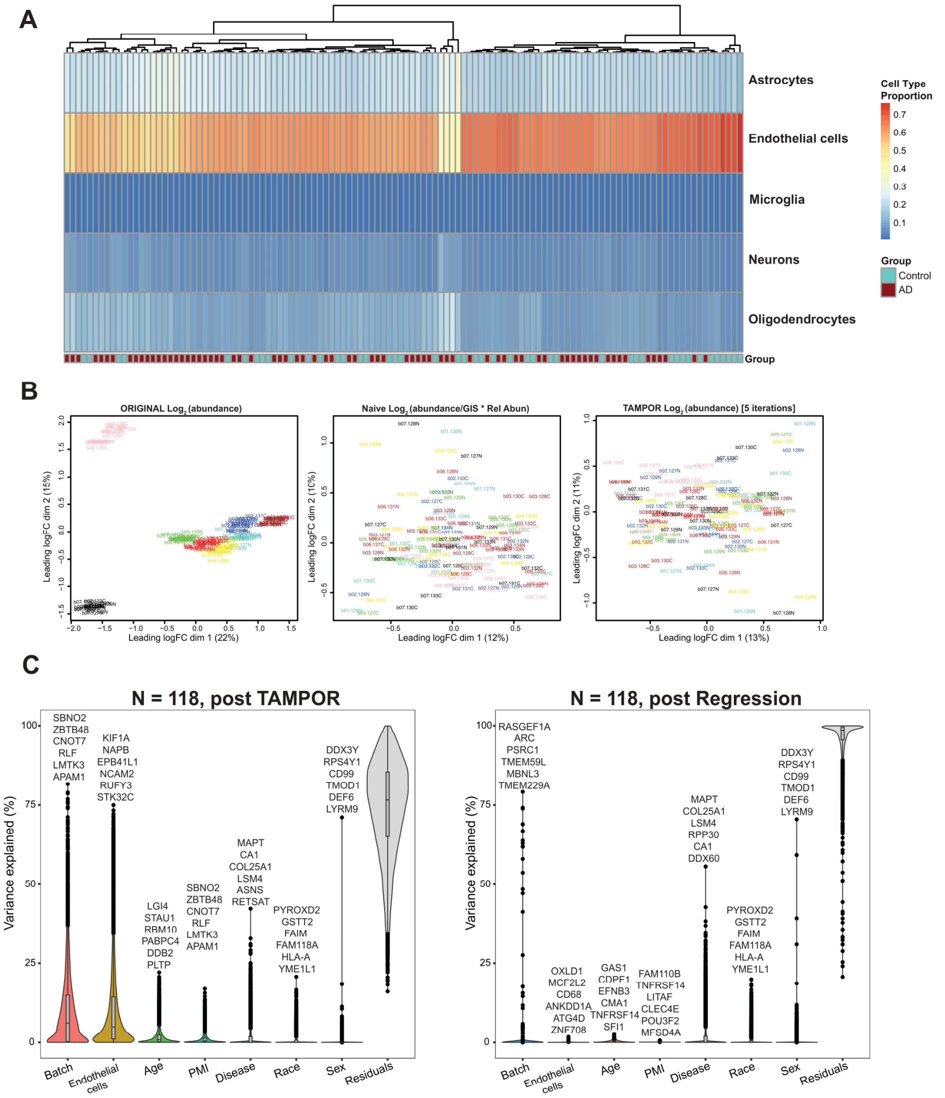

Figure S1. Assessment of cell type enrichment, batch effects, and co-variates on the cerebrovascular proteome.
(A) Heatmap illustrating core brain cell type contribution to cerebrovascular proteome. Proportions of the five cell types are defined via reprocessing of Darmanis, Nowakowski, and Zhong single-cell datasets as curated with the EnsDeconv R package, which calculated cell type proportions by ensemble deconvolution of cerebrovascular proteome. Dark red and orange indicate high enrichment of endothelial proteins to proportions above 70 percent, and dark blue indicates the lowest enrichment of cell-specific proteins in the preparations.
(B) Multidimensional scaling (MDS) illustrating TMT-MS batch correction. Log_2 abundance and log_2 abundance divided by the global internal standard (GIS) are shown.
(C) Variance partition plots were used to visualize the percent variance of each protein in the dataset co-varying with batch, endothelial cell type proportions, age, postmortem interval (PMI), disease, race, and sex. The matrix was subjected to bootstrap regression (right) to remove variance due to age, PMI, and endothelial cell type proportions.

medRxiv preprint doi: https://doi.org/10.1101/2025.10.08.25337413; this version posted October 16, 2025. The copyright holder for this preprint (which was not certified by peer review) is the author/funder, who has granted medRxiv a license to display the preprint in perpetuity.
It is made available under a CC-BY 4.0 International license .

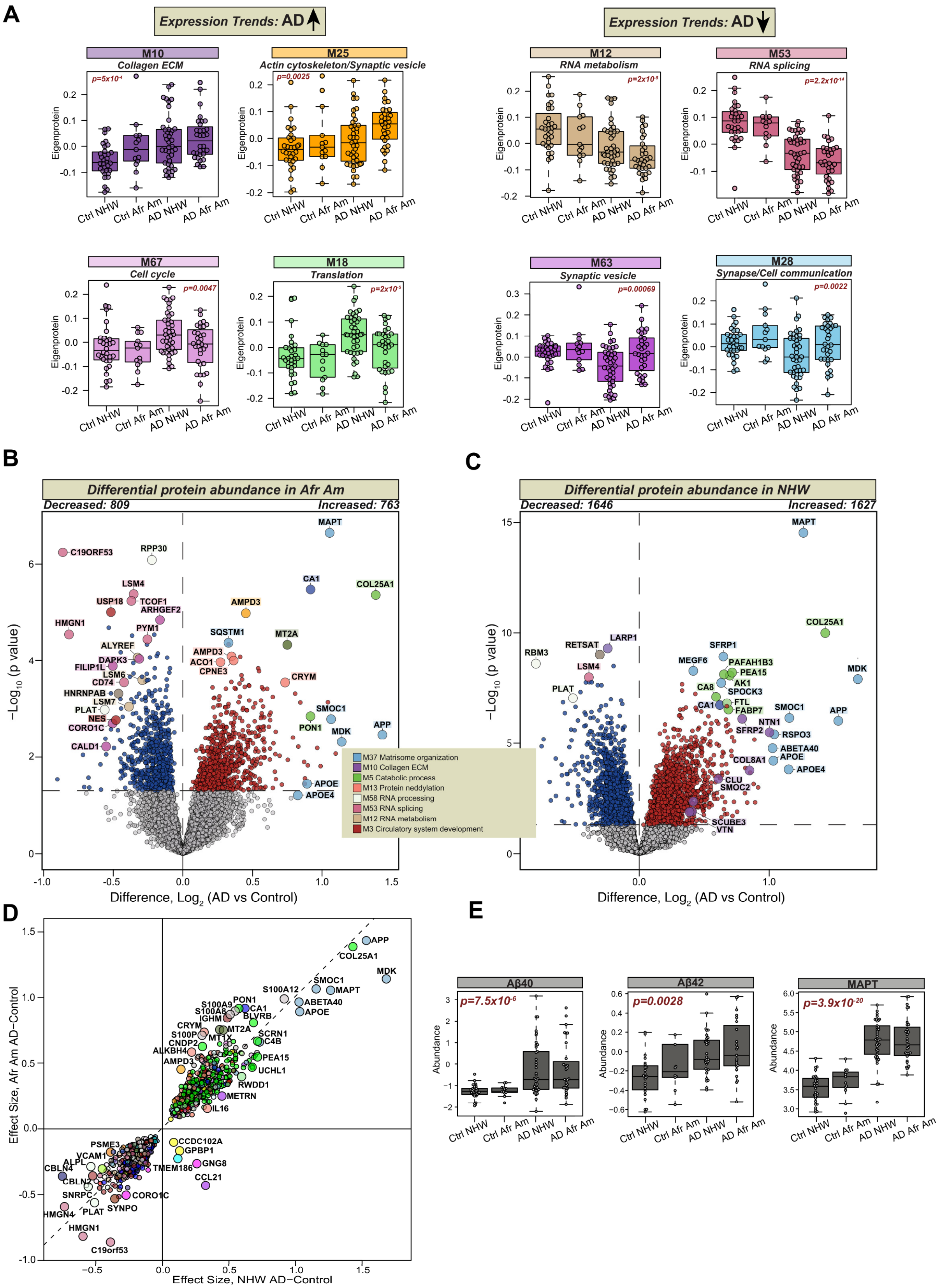

medRxiv preprint doi: https://doi.org/10.1101/2025.10.08.25337413; this version posted October 16, 2025. The copyright holder for this preprint (which was not certified by peer review) is the author/funder, who has granted medRxiv a license to display the preprint in perpetuity.
It is made available under a CC-BY 4.0 International license .

Figure S2. Race-specific cerebrovascular proteomic signatures in Alzheimer's Disease reveal distinct and shared pathways in African American and Non-Hispanic White individuals.
(A) Frontal cortical brain samples were collected from AD and control individuals (Emory: N= 11 African American (Afr Am), N= 9 Non-Hispanic Whites (NHW); UPenn: N=2 Afr Am, N=23 NHW) or Alzheimer's disease (Emory: N= 32 Afr Am, N= 29 NHW; UPenn: N=12). Module eigenprotein (ME) levels by race were presented for protein modules showing a significant change between groups. MEs were obtained by using one-way ANOVA test and p values are provided for each module. Box plots represent median, 25th and 75th percentile while box whiskers encompass actual data points up to 1.5 times the nearest interquartile range. Gene ontology analysis was used to assign a biological process to each module. (B-C) Volcano plots showing differential abundance of proteins measured in cerebrovasculature of African American (Afr Am) (b) or non-Hispanic White (NHW) (c), individuals between control and AD groups. The x axis illustrates the $\log_2$ fold change (AD vs. Control), while the y axis represents the $-\log_{10}$ statistical p value calculated for all proteins in each pairwise group, obtained as Tukey post-hoc test p values following one-way analysis of variance (ANOVA), except for imprecisely calculated Tukey values below $10^{-8.5}$ which underwent more precise and stringent Bonferroni post-hoc correction of a two-tailed unequal variance t-test. Proteins significantly elevated in Afr Am with AD (N=763) (b) or NHW (N=1627) (c) are shown in red whereas those significantly decreased in Afr Am AD (N = 809) (b) or NHW AD (N = 1646) (c) are depicted in blue ($p < 0.05$). Proteins of interest are shown as enlarged dots and shaded according to the color of their module membership.
(D) Scatterplot displaying a Pearson correlation between $\log_2$ effect size (AD vs. Control) of significantly altered proteins in cerebrovasculature of NHW and Afr Am cases. The significance of Pearson correlation was determined by Student's t-test for significance of correlation implemented in the WGCNA R package.
(E) TMT-MS quantified levels of amyloid-β (Aβ)40, Aβ42, and microtubule associated protein tau (MAPT) in cerebrovascular preparations across ethnoracial and diagnosis groups. Box plots represent median, 25th and 75th percentiles while box whiskers extend to non-outlier measurements up to 1.5 times each nearest interquartile range.

medRxiv preprint doi: https://doi.org/10.1101/2025.10.08.25337413; this version posted October 16, 2025. The copyright holder for this preprint (which was not certified by peer review) is the author/funder, who has granted medRxiv a license to display the preprint in perpetuity.
It is made available under a CC-BY 4.0 International license .

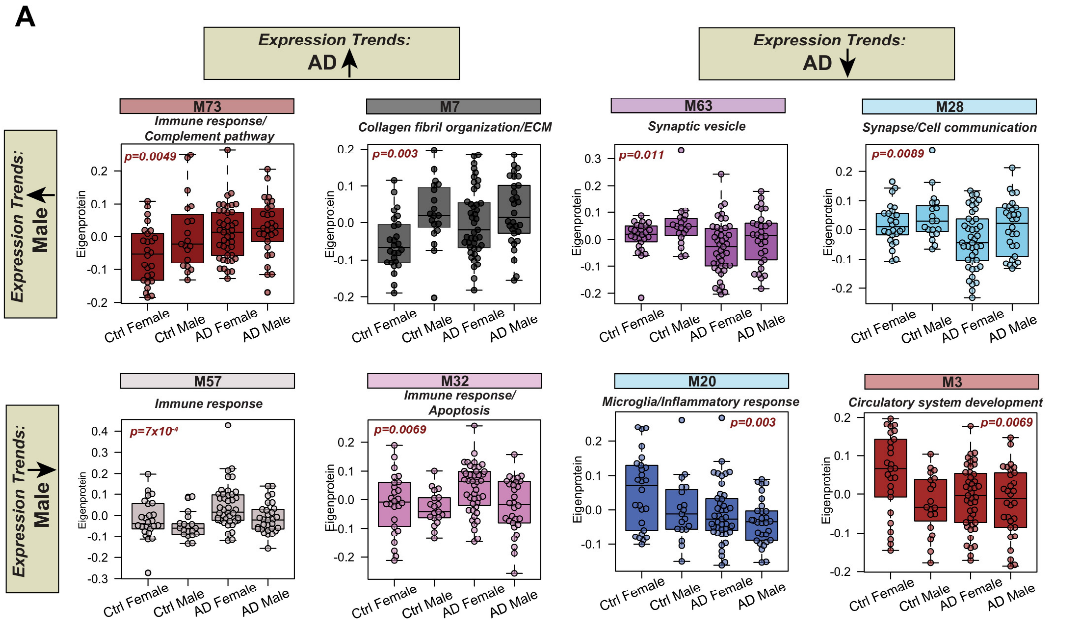

Figure S3. Sex- and diagnosis-associated variation in module abundance.
(A-B) Boxplots of selected modules display module eigenprotein level by sex (A) as well as sex and diagnosis (B). Box plots represent median, 25th and 75th percentile while box whiskers encompass actual data points up to 1.5 times the nearest interquartile range. Principal biology of each module was assessed by gene ontology analysis.

medRxiv preprint doi: https://doi.org/10.1101/2025.10.08.25337413; this version posted October 16, 2025. The copyright holder for this preprint (which was not certified by peer review) is the author/funder, who has granted medRxiv a license to display the preprint in perpetuity.
It is made available under a CC-BY 4.0 International license .

A

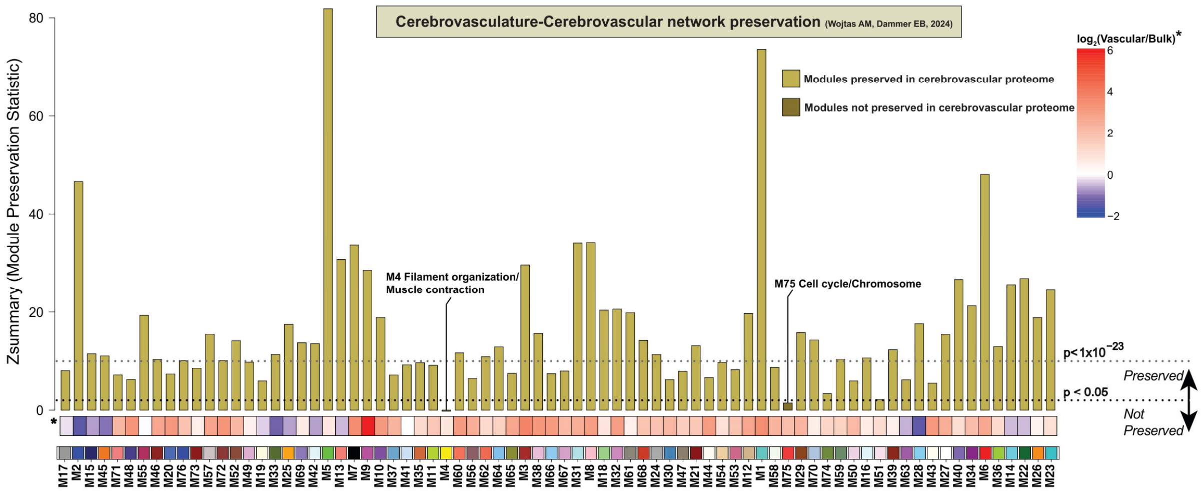

Figure S4. Modules associated with vascular biology are preserved in independent cerebrovascular proteome.
(A) Module preservation of the current TMT cerebrovascular network into the previously published cerebrovascular network (*Wojtas AM, Dammer EB, 2024*). $Z_{summary}$ score greater than or equal to 1.96 (or $p < 0.05$) indicated module preservation, whereas $Z_{summary}$ score less than 1.96 showed lack of preservation. Bar colors indicate the status of preservation.

medRxiv preprint doi: https://doi.org/10.1101/2025.10.08.25337413; this version posted October 16, 2025. The copyright holder for this preprint (which was not certified by peer review) is the author/funder, who has granted medRxiv a license to display the preprint in perpetuity.
It is made available under a CC-BY 4.0 International license .

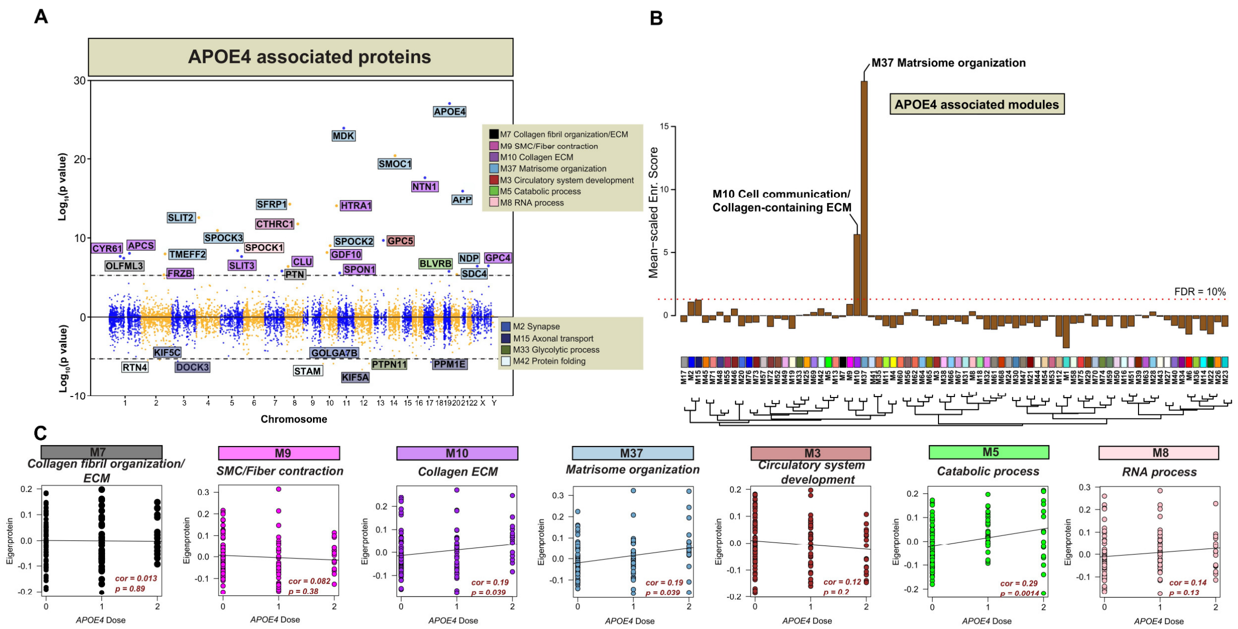

Figure S5. ***APOE*** **genotype is associated with extracellular matrix proteins.**
(A) 10,030 proteins measured in bulk tissue from ROSMAP cases (N=873). X-axis shows chromosome location for individual proteins and y-axis depicts positive and negative $\log_{10}$ *p values*. Proteins that survived Bonferroni correction are labeled. The colors indicate module membership in cerebrovascular network.
(B) Cerebrovascular module association to APOE4.
(C) Correlation plots of selected modules to *APOE4* dose (*APOE2/2,* N=1*; APOE2/3,* N=4*; APOE3/3,* N=44*; APOE2/4,* N=2*; APOE3/4,* N=36*; APOE4/4,* N=16). Correlation and p value are shown for each module.

medRxiv preprint doi: https://doi.org/10.1101/2025.10.08.25337413; this version posted October 16, 2025. The copyright holder for this preprint (which was not certified by peer review) is the author/funder, who has granted medRxiv a license to display the preprint in perpetuity.
It is made available under a CC-BY 4.0 International license .

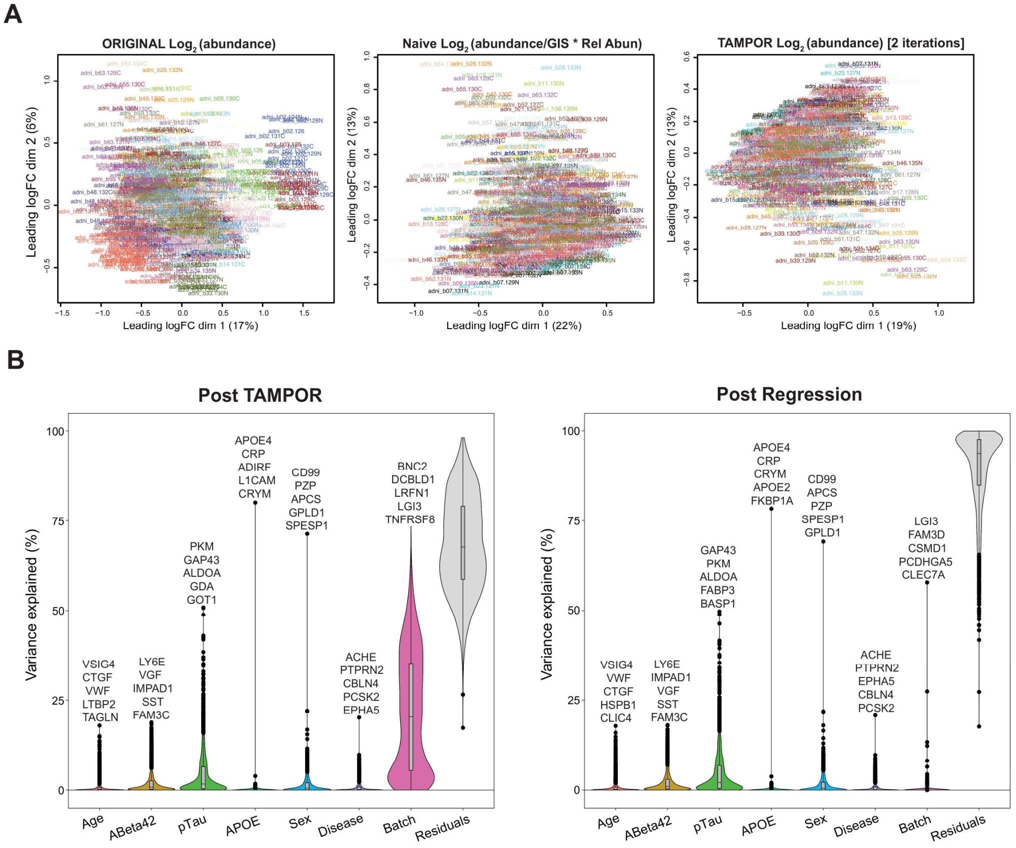

Figure S6. Quality control of the ADNI CSF proteome.
(A) Multidimensional scaling (MDS) illustrating TMT-MS batch correction. Log_2 abundance, log_2 abundance divided by the global internal standard (GIS), and TAMPOR are shown.
(B) Variance partition plots were used to visualize the percent variance of each protein in the dataset co-varying with batch, age and sex. The matrix was subjected to bootstrap regression (right) to remove variance due to batch.

medRxiv preprint doi: https://doi.org/10.1101/2025.10.08.25337413; this version posted October 16, 2025. The copyright holder for this preprint
(which was not certified by peer review) is the author/funder, who has granted medRxiv a license to display the preprint in perpetuity.
It is made available under a CC-BY 4.0 International license .

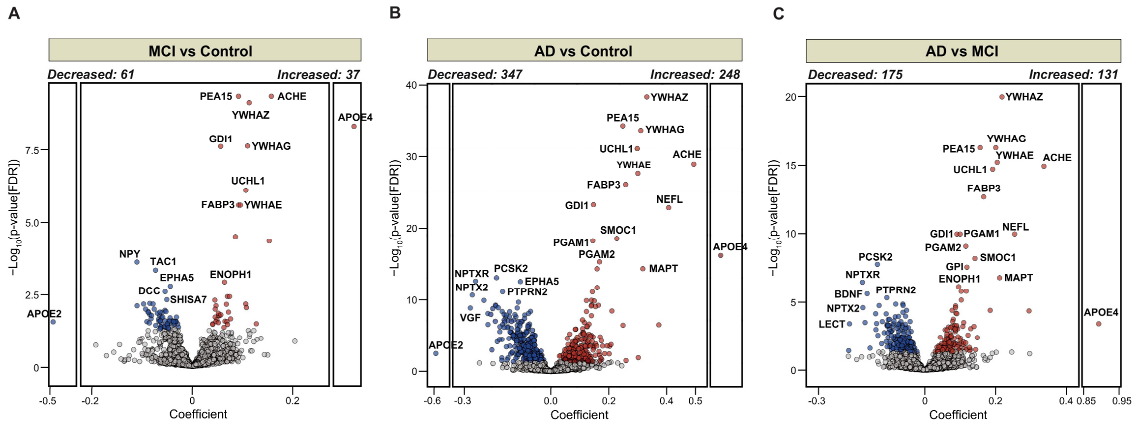

Figure S7. Differential abundance of CSF proteins in ADNI across clinical stages of dementia. (A-C) Volcano plots showing differential abundance of proteins at $P_{FDR}<0.05$ between clinically diagnosed control and MCI (Increased: 37, Decreased: 61) A), control and AD (Increased: 248, Decreased: 347) (B), and MCI and AD (Increased: 131, Decreased: 175) (C) (Controls: N=377, MCI: N=563, AD: N=164). The x axis represents the mean difference while the y axis shows the -$\log_{10}$ statistical *p* value calculated for all proteins in each group. *P* values were obtained from Student's t test. Proteins significantly increased are shown in red, whereas proteins significantly decreased are highlighted in blue. Proteins with unchanged levels are represented in grey.

medRxiv preprint doi: https://doi.org/10.1101/2025.10.08.25337413; this version posted October 16, 2025. The copyright holder for this preprint (which was not certified by peer review) is the author/funder, who has granted medRxiv a license to display the preprint in perpetuity.
It is made available under a CC-BY 4.0 International license .

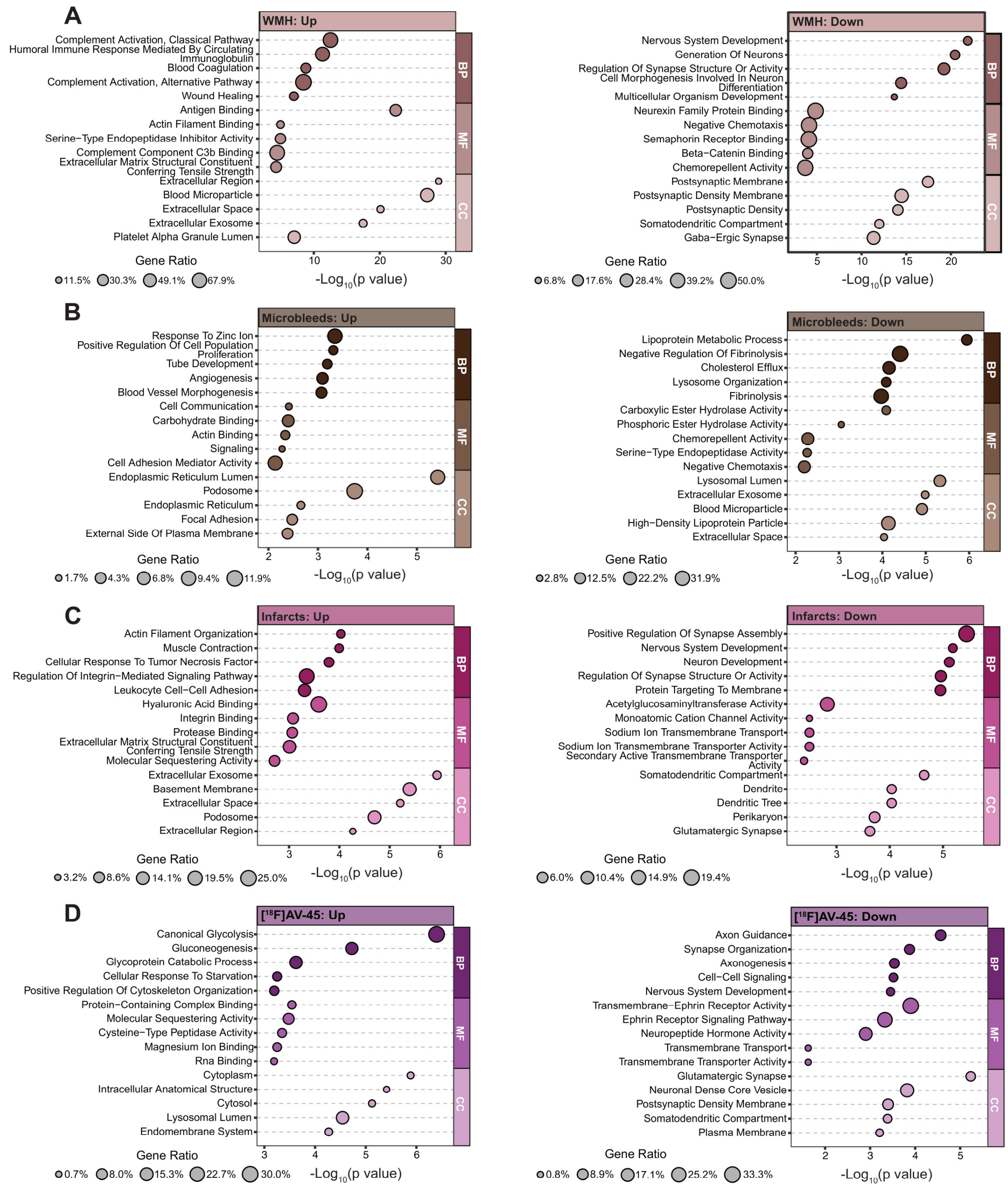

Figure S8. Biological processes underlying neuroimaging-based vascular abnormalities.
(A) Gene ontology analysis was performed to identify biological processes associated with each neurovascular manifestations (WMH, microbleeds, infarcts) as well as AV-45 using the proteins significantly associated with each imaging phenotype, as shown in Fig. 4. Upregulated and downregulated pathways are separately shown to highlight the directionality of the associations. Sample sizes were: WMH (N = 775), infarcts (N = 1,050), microbleeds (N = 732), and AV-45 (N = 745).

medRxiv preprint doi: https://doi.org/10.1101/2025.10.08.25337413; this version posted October 16, 2025. The copyright holder for this preprint (which was not certified by peer review) is the author/funder, who has granted medRxiv a license to display the preprint in perpetuity.
It is made available under a CC-BY 4.0 International license .

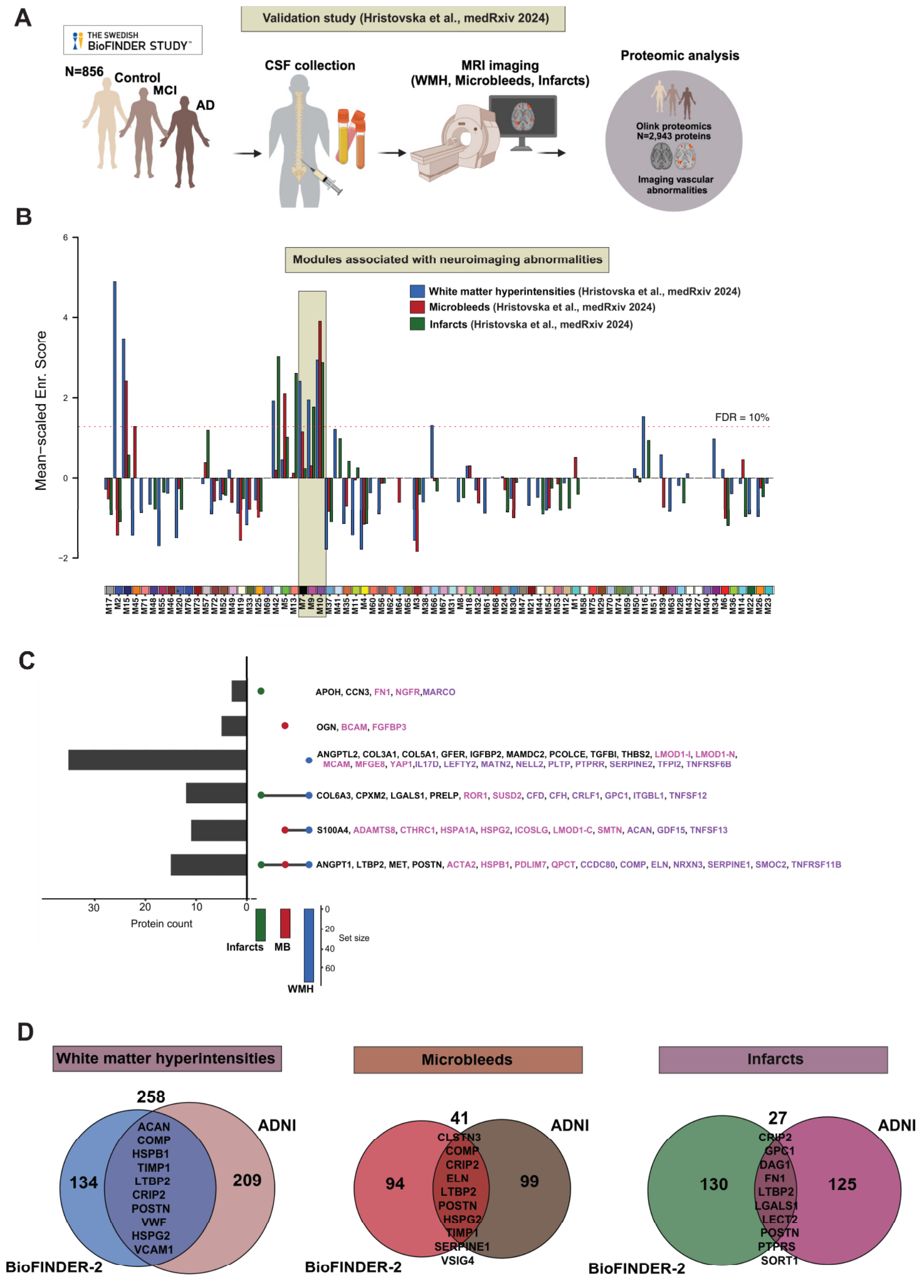

Figure S9. Cross-cohort validation of cerebrovascular protein modules linked to imaging markers and CAA severity in BioFINDER-2 and ADNI.
(A) Schematic representation of workflow in the Swedish BioFINDER study.
(B) Cerebrovascular network modules enriched for CSF proteins associated with imaging abnormalities from BioFINDER-2 cohort.
(C) UpSet plot illustrating proteins associated with WMH, microbleeds, and infarcts, highlighting the shared molecular signature across fluid-based markers of neurovascular injury in BioFINDER-2 cohort. (D) Venn diagrams showing the overlap between BioFINDER-2 and ADNI cohorts in the number of proteins associated with WMH, microbleeds, and infarcts. Top 10 overlapping proteins between datasets are shown in the boxes. An overlapping set of 886 proteins between ADNI and BioFINDER-2 was used in panels c and d, with significance defined as P<0.05 in both the ADNI and BioFINDER-2 cohorts.
